# Preoperative Sleep Disturbance Exaggerates Surgery-Induced Neuroinflammation and Neuronal Damage in Aged Mice

**DOI:** 10.1155/2019/8301725

**Published:** 2019-03-18

**Authors:** Pengfei Ni, Hongquan Dong, Qin Zhou, Yiwei Wang, Menghan Sun, Yanning Qian, Jie Sun

**Affiliations:** Department of Anesthesiology, The First Affiliated Hospital of Nanjing Medical University, 300 Guangzhou Road, Nanjing, Jiangsu 210029, China

## Abstract

Postoperative cognitive dysfunction (POCD) is defined as new cognitive impairment (memory impairment and impaired performance) after surgery, especially in aged patients. Sleep disturbance is a common phenomenon before surgery that has been increasingly thought to affect patient recovery. However, little is known about the functional impact of preoperative sleep disturbance on POCD. Here, we showed that tibial fracture surgery induced cognitive deficit and production of proinflammatory cytokines interleukin-6 (IL-6) and IL-1*β*, along with microglia and astrocyte activation, neuronal damage, and blood-brain barrier (BBB) disruption. Preoperative sleep disturbance enhanced the surgery-induced neuroinflammation, neuronal damage, BBB disruption, and memory impairment 24 h after surgery. Taken together, these results demonstrated that preoperative sleep disturbance aggravated postoperative cognitive function in aged mice and the mechanism may be related to central nervous system (CNS) inflammation and neuronal damage.

## 1. Introduction

Postoperative cognitive impairment is a rather prevalent clinical phenomenon that increases postoperative mobility and mortality; it is characterized by impairment of memory, comprehension, and attention function after surgery, especially in elderly patients [1–3]. Postoperative cognitive impairment reduces the quality of life and increases economic burdens for a growing number of old patients and is becoming more prevalent [4]. There is intense interest in the risk factors, including age, lower educational level, history of previous cerebral vascular accident, and preoperative cognitive impairment [1, 5], while little attention has been paid to sleep disruption before surgery.

Sleep disturbance is commonly observed before surgery and is even worse in older patients. Many studies suggested a close connection between sleep disturbance and learning and memory function [6–8]. Sleep optimizes consolidation of memory and promotes synaptic remodelling, and lack of sleep contributes to some neurodegenerative diseases [9–11]. For example, some studies have suggested that sleep drives *β*-amyloid clearance from the adult brain [12, 13] and *β*-amyloid is involved in the mechanism of Alzheimer's disease. Several clinical studies have shown that sleep problems after surgery could affect postoperative cognitive function [14, 15]. However, a number of patients also experience sleep problems before surgery; the association between preoperative sleep disturbance and cognitive impairment after surgery has scarcely been studied.

Neuroinflammation and neuronal damage are involved in multiple neurodegenerative diseases and are thought to play a critical role in the development of postoperative cognitive impairment [16–18]. The onset and progression of neuroinflammation rely on the activation of glial cells, mainly microglia and astrocytes. Activated glial cells release a host of proinflammatory and cytotoxic factors that are deleterious to neurons, in turn inducing further activation of glial cells and release of proinflammatory factors, resulting in a neuroinflammatory cascade [19–21]. In addition, BBB disruption may facilitate the movement of immune cells and proinflammatory factors from the periphery into the central nervous system, causing an increase in neuroinflammation and neuronal damage [22, 23].

Sleep disturbance can induce neuroinflammation, which may have an additive effect on cognitive impairment after surgery in aged mice. Therefore, the purpose of this study was to test the effect of preoperative sleep disturbance on the neuroinflammation and neuronal damage in POCD. We hypothesized that preoperative sleep disturbance would accelerate surgery-induced cognitive impairment by facilitating neuroinflammation, including inflammatory cytokine production and glial cell activation, neuronal damage, and BBB disruption.

## 2. Materials and Methods

### 2.1. Reagents

The rabbit anti-c-fos polyclonal antibody, rabbit anti-caspase-3 polyclonal antibody, rabbit anti-ionized calcium-binding adapter molecule 1 (Iba1) polyclonal antibody, and rabbit polyclonal anti-glial fibrillary acidic protein (GFAP) antibody were purchased from Abcam (Abcam, USA). The monoclonal mouse anti-claudin-5 antibody was purchased from Invitrogen (Invitrogen, USA). The monoclonal mouse anti-matrix metalloproteinase-2 (MMP-2), monoclonal mouse anti-MMP-9, and monoclonal rabbit anti-occludin antibodies were purchased from Abcam (Abcam, USA). RIPA buffer and the BCA kit were purchased from Beyotime (Shanghai, China). The mouse IL-6 ELISA kit and the IL-1*β* ELISA kit were obtained from R&D Systems (Minneapolis, MN, USA).

### 2.2. Animals

All experiments were performed on 15-month-old male wild-type C57BL/6J mice. Mice were kept in groups of five per cage and maintained in a standard environmental condition for the duration of the experiment (12 h light/dark cycle, lights on at 7:00 AM; ambient temperature, 22.0 ± 1.0°C; humidity, 40%±5%; and food and water available ad libitum). The experimental procedures followed the guidelines of the Institutional Animal Care and Use Committee of Nanjing Medical University and were approved by the Nanjing Medical University Animal Care and Use Committee.

### 2.3. Design and Treatment Groups

**Figure 1:**
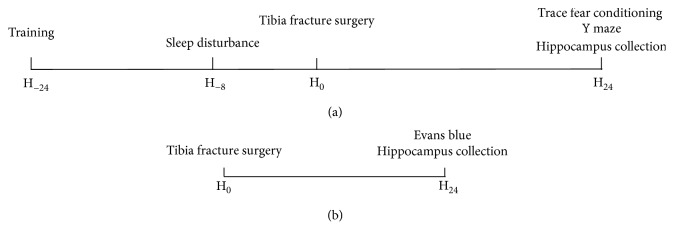
Study design. (a) Experiment 1: the training phase of the trace fear conditioning test was performed 1 day before surgery; afterwards, all mice were divided into four groups. Sleep disturbance lasted for 8 h before tibia fracture surgery. All mice underwent the trace fear conditioning and Y maze tests 24 h after surgery, after which the hippocampus was collected. (b) Experiment 2: after the behavioural tests, intravenous injection of EB was performed and the hippocampus was collected to detect EB extravasation.

The mice were randomly allocated to four groups with 12 mice in each group: (i) control group (Con group), (ii) sleep disturbance group (Sd group), (iii) surgery group (Sur group), and (iv) sleep disturbance before surgery group (Sd + Sur group). The mice in the Sd and Sd + Sur groups underwent sleep disturbance for 8 h, and the mice in the Sur and Sd + Sur groups underwent tibia fracture surgery after sleep disturbance. The study design is briefly illustrated in Figure 1.

### 2.4. Sleep Disturbance and Surgery

Sleep disturbance was initiated in the sleep disturbance and sleep disturbance before surgery groups at 7:00 AM and lasted 8 h. Sleep disturbance included directly observing the motor activity of the mice and gently stroking the fur with a brush when no activity was observed, as previously described [24]. Then, surgery was performed in the surgery and sleep disturbance before surgery groups after sleep disturbance at 3:00 PM. The surgically treated mice underwent an open tibia fracture, as previously described [25]. Briefly, following isoflurane anaesthesia (2.0% inspired concentration in 40% FiO_2_), the left tibia was shaved and disinfected and an incision was made anterolateral to the tibia, followed by an insertion of an intramedullary fixation pin. The soft tissues and periosteum were circumferentially stripped. Then, an osteotomy was created at the joint of the middle and the upper thirds of the tibia. Finally, the wound was disinfected. Throughout the surgical process, a warming pad was used to maintain an optimal temperature and analgesia (buprenorphine 0.1 mg/kg) was injected subcutaneously.

### 2.5. Behavioural Analyses

#### 2.5.1. Trace Fear Conditioning (TFC)

TFC has been used as a method to assess learning and memory in rodents. Before the conditioning procedure, mice were allowed to explore the environment for 100 s. Then, a conditioning stimulus (an auditory cue (65 dB, 3 kHz)) was presented for 20 s, followed by the unconditioned stimulus (a 0.5 s foot shock (0.7 mA)). The procedure was repeated after an interval of 100 s, and the mice remained there for another 30 s after the procedure. Finally, the mice were exposed to the same chamber again but without stimulus 24 h after surgery. Freezing behaviour was recorded for 300 s and was analysed with software (Xeye Fcs, Beijing Macro Ambition S&T Development Co. Ltd., Beijing, China).

#### 2.5.2. Y Maze

The Y maze was composed of three arms (regions I–III, 30 × 5 × 20 cm) with a lamp at the distal end and the arms converging to a common central area. The safe region was associated with the illumination, while the other regions featured electrical foot stimulation (25 V). Before the test, each mouse was placed randomly at the end of one arm and was allowed to explore the maze for 3 min to adapt to the environment. Then, the test was started and the illuminated arm was chosen to be the new starting area. Next, the orientation of the safe and stimulation regions was changed randomly. After each stimulation, the next stimulation was started until reaching the illuminated arm. A reaching time within 10 s was considered successful. If 9 responses were correct in 10 consecutive foot stimulations, the mice were considered to have satisfied the learning criterion. The total numbers that mice reached this criterion after stimulations during training were recorded as learning ability.

### 2.6. Immunohistochemistry

Mice were anaesthetized by 1% pentobarbital (10 *μ*l/g) and were perfused with 0.9% saline followed by cold 4% paraformaldehyde. The whole brains were harvested and fixed with 4% paraformaldehyde for 24 h at 4°C. Hippocampal sections (10 *μ*m thick) were prepared and fixed with 3% H_2_O_2_ in PBS for 10 min. They were then incubated in 10% bovine serum albumin with 0.3% Triton X-100 in 0.01 M phosphate-buffered saline for 1 h. An anti-Iba1 antibody (1 : 100, Abcam, USA) was used as a microglial marker, and the presence of astrocytes was identified using an anti-GFAP antibody (1 : 300, Abcam, USA). C-fos and caspase-3 expression was detected by incubation with the c-fos monoclonal antibody (1 : 300, Abcam, USA) and caspase-3 monoclonal antibody (1 : 1000, Abcam, USA) at 4°C overnight followed by incubation with an anti-rabbit secondary antibody for 2 h. Positive cells were visualized using DAB. Immunostaining images were digitally captured by using a Leica 2500 microscope.

### 2.7. ELISA

The levels of IL-6 and IL-1*β* in hippocampal tissue extracts were measured with an ELISA kit from R&D Systems. Briefly, samples were added to the microplates coated with monoclonal antibodies specific for mouse IL-6 or IL-1*β*. Then, wells were incubated at room temperature with test samples (hippocampal tissue extracts) and washed. This was followed by addition of conjugates of IL-6 and IL-1*β* individually and then incubation and washing again. Finally, the samples were incubated in substrate solution and the reaction was stopped with stop solution. Optical density values at 450 nm and 570 nm were measured and analysed using the Luminex-200 system version 2.3.

### 2.8. Evans Blue (EB) Extravasation

To evaluate BBB permeability, mice were injected intravenously with 2% EB (Sigma-Aldrich) at a dose of 4 ml/kg. One hour after the injection, the mice were anaesthetized and perfused with normal saline (20 ml) through the left ventricle. Hippocampal tissue was extracted and immersed in formamide (Sigma-Aldrich) at 37°C for 72 h. Then, formamide was centrifuged at 12000 *g* for 20 min. The absorbance of was measured at 632 nm (BioTek, Winooski, Vermont, USA), and the EB content was calculated from the standard EB curve to measure BBB permeability.

### 2.9. Western Blotting

Hippocampal tissues were homogenized in RIPA lysis buffer and centrifuged for 20 min at 12000 *g* (4°C). Then, the supernatants were harvested for immunoblot analysis and the protein concentration was measured by the BCA kit. The protein samples were denatured with sodium dodecyl sulfate (SDS) sample buffer, separated by 10% SDS polyacrylamide gel electrophoresis and transferred onto polyvinylidene fluoride (PVDF) membranes (Millipore). Subsequently, the membranes were blocked with 5% skim milk at room temperature for 1 h and were incubated with corresponding antibodies overnight at 4°C. The following primary antibodies were used: monoclonal rabbit anti-Iba1 (1 : 1000, Abcam, USA), monoclonal rabbit anti-GFAP (1 : 500, Abcam, USA), monoclonal rabbit anti-albumin (1 : 1000, Invitrogen, USA), monoclonal rabbit anti-occludin (1 : 1000, Abcam, USA), monoclonal mouse anti-claudin-5 (1 : 500, Invitrogen, USA), monoclonal rabbit anti-MMP-2 (1 : 1000, Invitrogen, USA), and monoclonal rabbit anti-MMP-9 (1 : 500, Abcam, USA). After incubation with an anti-rabbit or anti-mouse secondary antibody (1 : 5000) for 1 h, the protein on the membranes was finally visualized with an ECL kit (Thermo Fisher Scientific, Rockford, IL, USA).

### 2.10. Statistical Analysis

All data were first tested for normality by the Shapiro–Wilk test and for homoscedasticity by Levene's test. All data are expressed as the mean ± sem. The significance of the differences between groups was determined by one-way ANOVA followed by either the post hoc least significant difference test (the variance was equal) or Dunnett T3 test (the variance was not equal). *P* < 0.05 was considered statistically significant.

## 3. Results

### 3.1. Preoperative Sleep Disturbance Enhanced Postoperative Cognitive Impairment in Aged Mice

**Figure 2:**
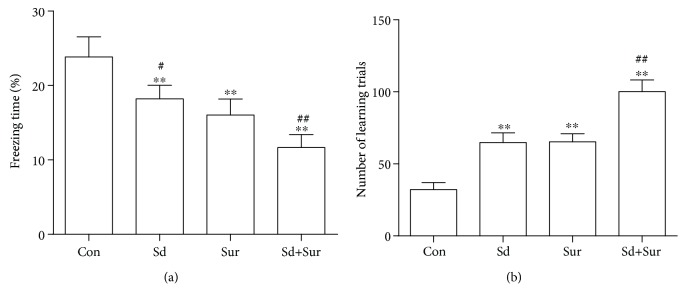
Preoperative sleep disturbance enhanced postoperative cognitive impairment in aged mice. (a) The freezing time in the contextual fear response task. (b) The number of learning trials in the Y maze test. The data are presented as the mean ± sem (*n* = 12). ^∗∗^*P* < 0.01 vs. the Con group; ^#^*P* < 0.05 and ^##^*P* < 0.01 vs. the Sur group.

To examine whether preoperative sleep disturbance enhanced postoperative cognitive decline, we assessed mouse freezing time via the TFC test and the number of learning trials on the Y maze test. Compared with controls, mice in the surgery group had less freezing behaviour and more learning trials, indicating impaired cognitive function. In addition, in the sleep disturbance before surgery group, mice showed a more notable reduction in freezing behaviour and increased learning trials compared with those in the surgery group (Table 1) and (Figure 2). These results suggested that preoperative sleep disturbance aggravated postoperative cognitive impairment in aged mice.

### 3.2. Preoperative Sleep Disturbance Promoted the Surgery-Induced Increase of IL-6 and IL-1*β* Levels in the Hippocampus

**Figure 3:**
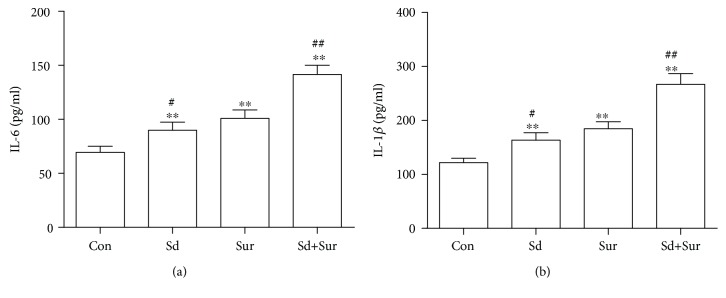
Preoperative sleep disturbance promoted the surgery-induced increase of IL-6 and IL-1*β* levels in the hippocampus. (a) Levels of IL-6 in the hippocampus as detected by ELISA. (b) Levels of IL-1*β* in the hippocampus as detected by ELISA. The data are presented as the mean ± sem (*n* = 6). ^∗∗^*P* < 0.01 vs. the Con group; ^#^*P* < 0.05 and ^##^*P* < 0.01 vs. the Sur group.

Since neuroinflammation was closely related to cognitive decline after surgery, the levels of the proinflammatory cytokines IL-6 and IL-1*β* in the hippocampus were examined 24 h after tibial fracture surgery by ELISA. Levels of the proinflammatory cytokines IL-6 and IL-1*β* in the sleep disturbance group and the surgery group were higher than those in the control group. In addition, we observed a significant increase in the IL-6 and IL-1*β* levels in the sleep disturbance before surgery group compared with the surgery alone group (Figure 3). These results suggested that sleep disturbance before surgery accelerated the surgery-induced release of the proinflammatory cytokines IL-6 and IL-1*β*.

### 3.3. Preoperative Sleep Disturbance Enhanced Microglial Activation in the Hippocampus Induced by the Tibial Surgery

**Figure 4:**
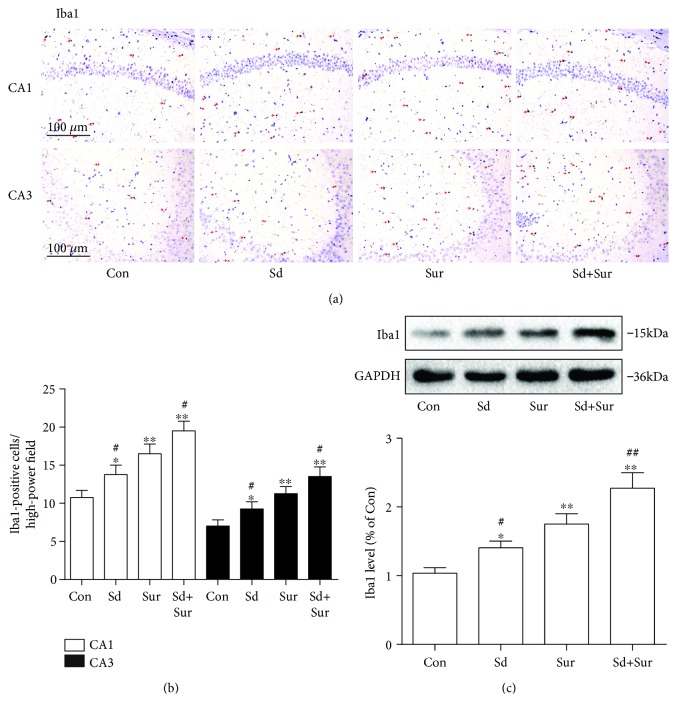
Preoperative sleep disturbance enhanced microglial activation in the hippocampus induced by the tibial surgery. (a) Representative immunohistochemistry images of microglia (red arrows) in areas CA1 and CA3 of the hippocampus based on staining with an anti-Iba1 antibody. Scale bar: 100 *μ*m. (b) Quantification of Iba1-positive cells in areas CA1 and CA3 of the hippocampus. (c) Expression levels of Iba1 protein in the hippocampus. The data are presented as the mean ± sem (*n* = 4). ^∗^*P* < 0.05 and ^∗∗^*P* < 0.01 vs. the Con group; ^#^*P* < 0.05 and ^##^*P* < 0.01 vs. the Sur group.

Microglia play an important role in the development of neuroinflammation in several central nervous system diseases. Therefore, to assess microglial activation, we detected expression of Iba1, a marker of microglia, in areas CA1 and CA3 of the hippocampus by immunostaining. Mice in the surgery group showed more Iba1-positive cells in hippocampal tissue than did the control group, and preoperative sleep disturbance led to a notable increase in microglial activation compared to surgery alone. In addition, the microglia displayed enlarged cell bodies and irregular shapes, in accordance with the morphology of activated microglia (Figures 4(a) and 4(b)). In addition, we detected the protein expression of Iba1 in the hippocampus by Western blotting and the results were accordance with the above findings (Figure 4(c)). These data suggested that preoperative sleep disturbance accelerated microglial activation induced by surgery.

### 3.4. Preoperative Sleep Disturbance Accelerated Surgery-Induced Astrocyte Activation in the Hippocampus

**Figure 5:**
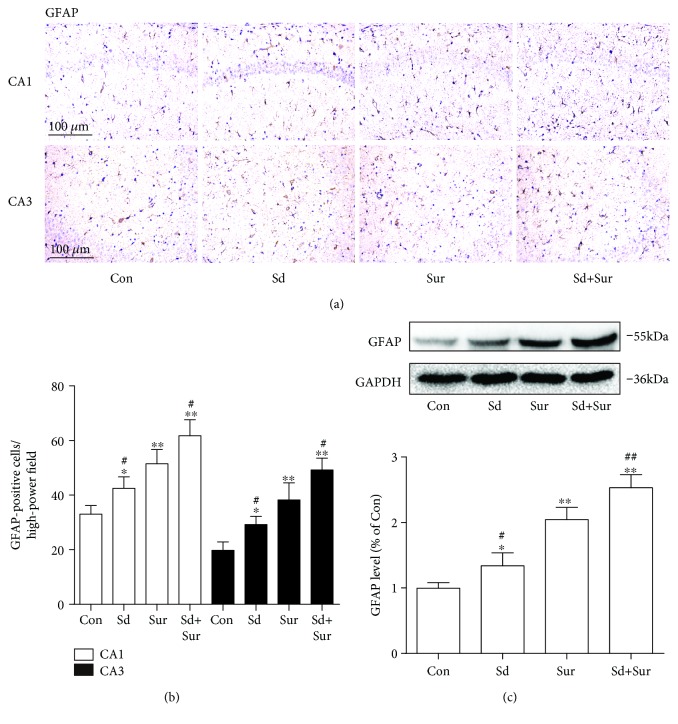
Preoperative sleep disturbance accelerated surgery-induced astrocyte activation in the hippocampus. (a) Representative images of activated astrocytes in areas CA1 and CA3 of the hippocampus based on staining with an anti-GFAP antibody. Scale bar: 100 *μ*m. (b) Quantification of GFAP-positive cell areas CA1 and CA3 of the hippocampus. (c) Expression levels of GFAP protein. The data are presented as the mean ± sem (*n* = 4). ^∗^*P* < 0.05 and ^∗∗^*P* < 0.01 vs. the Con group; ^#^*P* < 0.05 and ^##^*P* < 0.01 vs. the Sur group.

Astrocytes, one of the main glial cells, participate in not only neuronal apoptosis but also neuroinflammation. To explore the effect of surgery and preoperative sleep disturbance on astrocyte activation, we detected the expression of GFAP, a marker of activated astrocytes, in areas CA1 and CA3 of the hippocampus by immunostaining. The number of GFAP-positive cells was increased 24 h after surgery compared to the control levels. Moreover, sleep deprivation before surgery induced further increases in the number of activated astrocytes compared to surgery alone (Figures 5(a) and 5(b)). In addition, we detected protein expression of GFAP in the hippocampus by Western blotting and the results were in accordance with the above findings (Figure 5(c)). These results indicated that preoperative sleep deprivation aggravated astrocyte activation induced by surgery.

### 3.5. Preoperative Sleep Disturbance Augmented Surgery-Induced Neuronal Activation and Apoptosis in the Hippocampus

**Figure 6:**
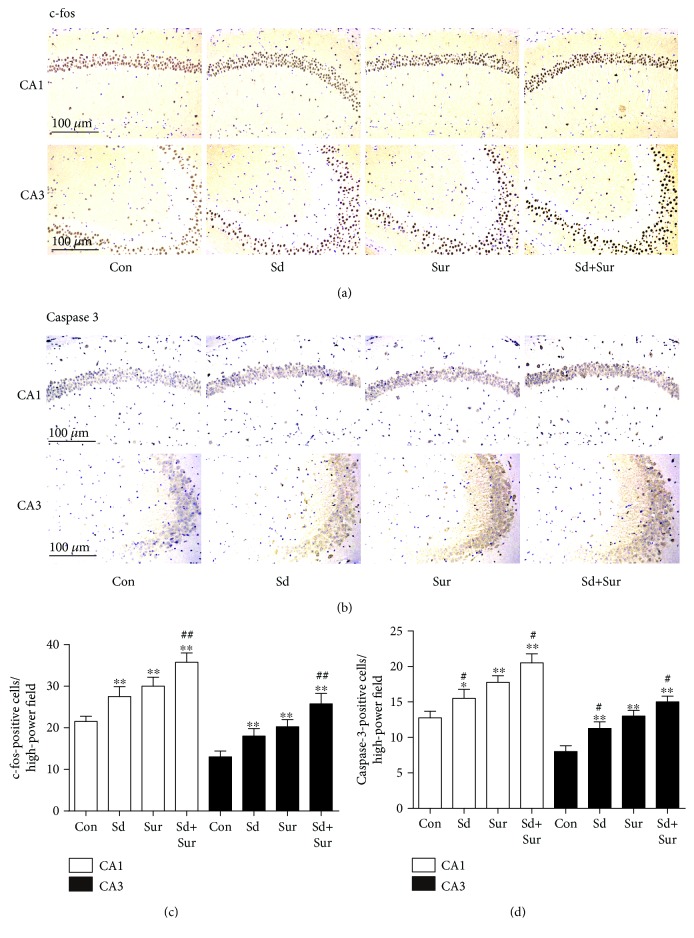
Preoperative sleep disturbance augmented surgery-induced neuronal activation and apoptosis in the hippocampus. (a, b) Representative immunohistochemistry images of neuronal damage based on staining with anti-c-fos and anti-caspsase-3 antibody in areas CA1 and CA3 of the hippocampus. Scale bar: 100 *μ*m. (c, d) Quantification of c-fos-positive and caspsase-3-positive cells in areas CA1 and CA3 of the hippocampus. The data are presented as the mean ± sem (*n* = 4). ^∗^*P* < 0.05 and ^∗∗^*P* < 0.01 vs. the Con group; ^#^*P* < 0.05 and ^##^*P* < 0.01 vs. the Sur group.

To examine whether preoperative sleep disturbance had an effect on neurons in POCD, we detected the expression of c-fos, widely considered to be a marker of neuronal activation, in areas CA1 and CA3 of the hippocampus by immunohistochemistry. As shown in Figures 6(a) and 6(c), c-fos expression in the hippocampus in the surgery group was substantially higher than that in the control group and sleep disturbance before surgery led to significantly more c-fos expression than did surgery alone. Furthermore, we examined neuronal apoptosis by detecting caspase-3 expression in areas CA1 and CA3 of the hippocampus by immunohistochemistry. Levels of neuronal apoptosis in the sleep disturbance and surgery groups were higher than those of the control group; we also observed a more substantial increase in neuronal apoptosis in the sleep disturbance before surgery group than in the surgery group (Figures 6(b) and 6(d)). These results suggested that sleep disturbance before surgery aggravated surgery-induced neuronal activation and apoptosis.

### 3.6. Preoperative Sleep Disturbance Augmented Surgery-Induced BBB Disruption

**Figure 7:**
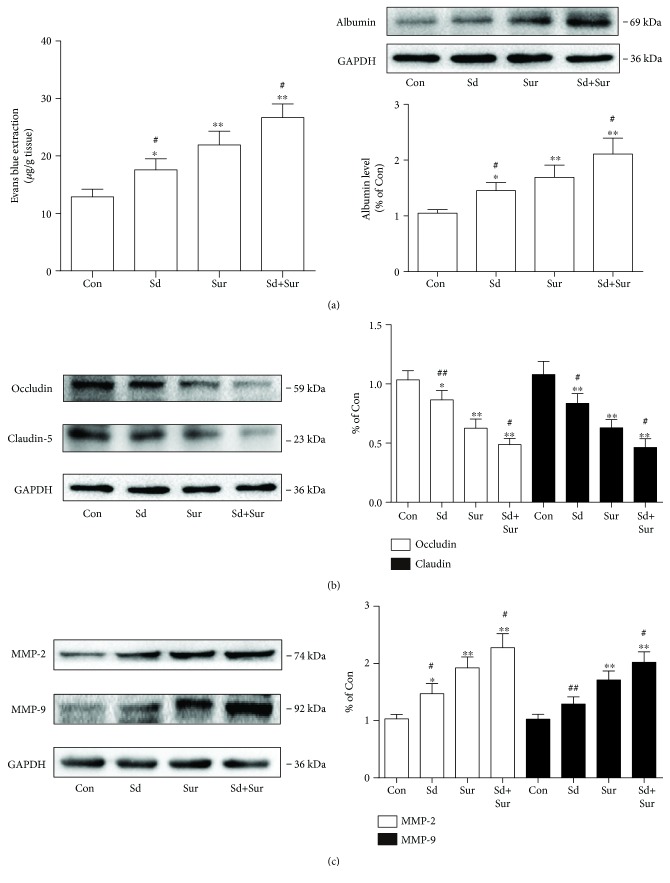
Preoperative sleep disturbance augmented operation-induced blood-brain barrier disruption. (a) EB content and the protein levels of albumin in the hippocampus. (b) Expression levels of occludin and claudin-5 protein in the hippocampus. (c) Expression levels of MMP-2 and MMP-9 protein in the hippocampus. The data are presented as the mean ± sem (*n* = 4). ^∗^*P* < 0.05 and ^∗∗^*P* < 0.01 vs. the Con group; ^#^*P* < 0.05 and ^##^*P* < 0.01 vs. the Sur group.

The BBB is a crucial structure for maintaining CNS stability. Recent findings suggested that surgery disrupts the BBB, contributing to neuroinflammation and cognitive dysfunction. To investigate the impact of surgery and preoperative sleep disturbance on the BBB, we detected BBB permeability by EB extravasation and examined the level of albumin in the hippocampus. In addition, to assess BBB permeability, we tested the levels of occludin and claudin-5, key components of tight junctions (TJs), in the hippocampus 24 h after surgery. The EB content and albumin levels were significantly higher in the surgery group than in the control group, and the sleep disturbance before surgery group showed more substantial increases than did the surgery group (Figure 7(a)). In contrary to the above results, levels of occludin and claudin-5 were substantially lower after surgery and preoperative sleep disturbance produced further reductions (Figure 7(b)). Since MMPs can disrupt the extracellular matrix and influence the integrity of the BBB, we evaluated the expression of MMP-2 and MMP-9 in the hippocampus. Surgery induced increased expression of MMP-2 and MMP-9, and the degree of these increases was greater in the sleep disturbance before surgery group (Figure 7(c)). These results suggested that preoperative sleep disturbance enhanced surgery-induced BBB disruption.

## 4. Discussion

The underlying pathological mechanism of neuroinflammation and neuronal damage may reveal an approach to the treatment of many central nervous system disorders. Some studies explored the neuroinflammatory and neuronal damage mechanisms of sleep disturbance and surgery separately but overlooked the interaction between the two. Given the strong connection between sleep disturbance and surgery in clinical conditions, we explored the effect of sleep disturbance before surgery on cognitive decline induced by surgery. We used a sleep disturbance model and an open tibia fracture model as previously described [24, 25], and we found that preoperative sleep disturbance increased the extent of surgery-induced microglial activation, astrocyte activation, and BBB dysfunction, which aggravate neuroinflammation and neuronal damage.

Studies of postoperative cognitive decline suggested that surgery may induce impairment of exploratory behaviour and spatially based working memory, especially in older individuals [26]. Numerous studies have also shown that sleep disturbance may impair neuronal plasticity and formation and consolidation of memories, resulting in learning and memory impairments, such as poor decision making and impaired attention and learning [27]. Complex attention and working memory could change following one night without sleep [28]. Interestingly, cognitive decline in both cases is closely related to the hippocampus [29, 30]. TFC is a widely used method to assess learning and memory function in rodents [31]. In this study, we found that surgery induced impairment in exploratory behaviour in old C57BL/6 wild-type mice, which was accelerated by 8-hour sleep disturbance before surgery. The results were further confirmed by the Y maze test, a paradigmatic test of spatially based working memory [32]. These two methods are usually used to assess learning and memory function that is critically dependent on the hippocampus. The results of the Y maze test were approximately in accordance with those of the TFC task.

Emerging evidence suggests a significant role for neuroinflammation in the occurrence and development of neurodegenerative diseases [33–35]. Various clinical data and animal studies showed correlations between inflammation and cognitive impairment after surgery [36, 37]. The immune responses in the brain are regulated by the microglia and astrocytes, as well as BBB. What is more, some studies revealed that surgery induced activation of microglia and astrocytes and disruption of the BBB, resulting in neuroinflammation and impairment of learning and memory [38]. Microglia and astrocyte activation not only leads to the production of neurotoxic factors, such as tumor necrosis factor-*α* (TNF-*α*), IL-1*β*, and IL-6, but also generates reactive oxygen and nitrogen species (ROS/RNS), which may potentiate neuronal damage [39, 40]. Our previous work showed that the neuroinflammation reaches a peak 24 h after surgery [41]. Therefore, in this study, we chose 24 h after surgery as the time point for observations.

Sleep plays a crucial role in the immune system, and sleep disturbance was reported to contribute to neuroinflammation and neuronal dysfunction [42, 43]. Sleep loss was reported to be associated with increases in cytokine levels [44]. Besides, sleep loss could promote astrocytic phagocytosis and microglial activation in the cerebral cortex and impair blood-brain barrier function [45–47]. However, the experimental subjects of these sleep disturbance studies are commonly in normal healthy conditions. Subjects undergoing surgical stress may be more vulnerable to the adverse effects of sleep disturbance. Sleep problems are common before surgery, but there is a lack of research focusing on the effects of preoperative sleep disturbance. In this study, we found that surgery induced cytokine secretion, microglia and astrocyte activation, neuronal damage, and BBB disruption. Moreover, preoperative sleep disturbance enhanced surgery-induced neuroinflammation, neuronal activation, and apoptosis, which resulted in the enhanced impairment of learning and memory. Furthermore, we found that surgery induced BBB disruption, manifesting as decreased levels of occludin and claudin-5 and increased levels of MMP-2 and MMP-9. We also observed that preoperative sleep disturbance enhanced surgery-induced BBB disruption. These results confirmed that sleep disturbance before surgery exacerbated surgery-induced neuroinflammation and neuronal damage in the hippocampus of aged mice.

One of the limitations of this study is that the sleep was not recorded in the mice. As demonstrated in some studies with electroencephalogram (EEG) recordings, sleep/wake activity can be measured by behavioural state assessment. To define sleep as 50 seconds or more of continuous inactivity has an 88–94% agreement with EEG definition [24]. Thus, the method used in this study was effective in recording sleep/wake activity. Besides, the lack of EEG recording instrumentation avoids the possible impact of surgical implantation on the results. However, without EEG recording, the different stages of sleep deprivation could not be further explored in this study. Another limitation is that there was no age comparison group in this study (young vs. aged mice). Aging has been associated with diverse changes in sleep, including decreased total sleep time and sleep efficiency and increased sleep fragmentation [48]. Besides, aging is a risk factor for postoperative cognitive impairment and the response to surgery stress may differ between young and aged mice [49]. In this study, we mainly focused on the impact of acute sleep disturbance on POCD. Some clinical research suggested that chronic impaired sleep predicts cognitive decline in old people [50]. Sleep disturbance is common in mild cognitive impairment (MCI), and this may reveal an ongoing neurodegenerative process [51]. Thus, investigating the effect and predicative functions of chronic impaired sleep disturbance on postoperative neurocognitive disease is an important future direction.

## 5. Conclusions

In summary, this study suggested that peripheral surgery induced inflammatory cytokine production, glial cell activation, neuronal damage, and BBB disruption, resulting in postoperative cognitive dysfunction. Moreover, these effects were exacerbated by acute sleep disturbance before surgery. The exact role of acute preoperative sleep disturbance on POCD and the underlying mechanisms may provide novel ideas for prevention or mitigation of cognitive impairment after surgery and may have implications for preoperative preparation.

## Figures and Tables

**Table 1:** Analysis of freezing time in TFC and number of learning trails in the Y maze test.

Group (*n* = 12)	Con	Sd	Sur	Sd + Sur
TFC: freezing time (%)	23.83 ± 0.78	18.13±0.49^∗∗^, ^#^	16.03±0.62^∗∗^	11.66±0.51^∗∗^, ^##^
Y maze: number of learning trails	32.17 ± 1.40	64.83±1.93^∗∗^	65.25±1.65^∗∗^	100.20±2.34^∗∗^, ^##^

All values are expressed as mean ± sem. ^∗∗^*P* < 0.01 vs. the Con group; ^#^*P* < 0.05 and ^##^*P* < 0.01 vs. the Sur group.

## Data Availability

The data used to support the findings of this study are available from the corresponding author upon request.

## Abbreviations

POCD: Postoperative cognitive dysfunction
TFC: Trace fear conditioning
IL-6: Interleukin-6
IL-1*β*: Interleukin-1*β*
BBB: Blood-brain barrier
CNS: Central nervous system
Iba1: Ionized calcium-binding adapter molecule 1
GFAP: Glial fibrillary acidic protein
MMP-2: Matrix metalloproteinase-2
MMP-9: Matrix metalloproteinase-9
EB: Evans blue
SDS: Sodium dodecyl sulfate
PVDF: Polyvinylidene fluoride.
